# Mammalian TRAPPIII Complex positively modulates the recruitment of Sec13/31 onto COPII vesicles

**DOI:** 10.1038/srep43207

**Published:** 2017-02-27

**Authors:** Shan Zhao, Chun Man Li, Xiao Min Luo, Gavin Ka Yu Siu, Wen Jia Gan, Lin Zhang, William K. K. Wu, Hsiao Chang Chan, Sidney Yu

**Affiliations:** 1 School of Biomedical Sciences, The Chinese University of Hong Kong, Sha Tin, N.T., Hong Kong SAR, P.R. China; 2 Department of anesthesia, Faculty of Medicine, The Chinese University of Hong Kong, Shatin, N.T., Hong Kong SAR, P.R. China; 3 Epithelial Cell Biology Research Centre, The Chinese University of Hong Kong, Sha Tin, N.T., Hong Kong SAR, P.R. China

## Abstract

The Transport protein particle (TRAPP) complex is a tethering factor for COPII vesicle. Of three forms of TRAPP (TRAPPI, II and III) complexes identified so far, TRAPPIII has been largely considered to play a role in autophagy. While depletion of TRAPPIII specific subunits caused defects in the early secretory pathway and TRAPPIII might interact with components of the COPII vesicle coat, its exact role remains to be determined. In this study, we studied the function of TRAPPIII in early secretory pathway using a TRAPPIII-specific subunit, TRAPPC12, as starting point. We found that TRAPPC12 was localized to the ER exit sites and ERGIC. In cells deleted with TRAPPC12, ERGIC and to a lesser extent, the Golgi became dispersed. ER-to-Golgi transport was also delayed. TRAPPC12, but not TRAPPC8, bound to Sec13/Sec31A tetramer but each Sec protein alone could not interact with TRAPPC12. TRAPPIII positively modulated the assembly of COPII outer layer during COPII vesicle formation. These results identified a novel function of TRAPPIII as a positive modulator of the outer layer of the COPII coat.

Vesicular traffic from the ER to Golgi complex requires the sequential action of two different coat complexes, COPII and COP I^1^. These coat complexes are needed for cargo selection and vesicle budding. The COPII vesicle is assembled on a specialized region of the ER membranes called the ER exit sites (ERES)^2^. The coat for COPII vesicles contains five proteins: Sar1, Sec23/Sec24, Sec13/Sec31. The assembly of the COPII coat occurs in a stepwise fashion, beginning with the recruitment of the GTPase Sar1 through GTP loading facilitated by its GEF (guanine nucleotide exchange factor)^3^. Sar1-GTP subsequently recruits one heterodimer of Sec23/24 through the interaction between Sec23 to Sar1-GTP. Sar1-Sec23/Sec24-cargoes, referred to as “pre-budding complex”, represent a basic functional unit of the COPII inner coat layer. Following pre-budding complex formation, tetrameric Sec13/Sec31 is recruited via the interaction between Sec23 and Sec31. The binding of Sec13/Sec31 forms the outer layer of COPII coat.

The tethering of COPII vesicle at the Golgi membrane surface was mediated by a protein complex called TRAPP (Transport protein particle)^4^. Subsequently, at least three forms of TRAPP complexes (TRAPPI, II and III) have been identified and vesicle tethering function has been assigned to TRAPPI. The interaction between Bet3(TRAPPC3) and Sec23 was thought to mediate tethering^5^. Structurally, TRAPPI contains six subunits, Bet5p, Trs20p, two copies of Bet3p, Trs23p, Trs31p and Trs33p in yeast. Their mammalian homologs are designated as TRAPPC1 to TRAPPC6 respectively. TRAPPII contains all the subunits of TRAPPI plus additional subunits including Trs120p/TRAPPC9 and Trs130p/TRAPPC10, Trs65p/TRAPPC13 and Tcap17p(TRAPPC2L)^6^,^7^,^8^,^9^. Electron microscopy (EM) structure of this complex showed that yeast TRAPPII is a dimer^10^. TRAPPIII contains TRAPPI core subunit plus Trs85 in yeast but in mammalian cells, TRAPPIII contained TRAPPI core and TRAPPC8 and perhaps subunits unique in mammals (see result section and refs 11, 12, 13).

TRAPPC12, also called CGI-87, TTC15 and TRAMM, has been recently identified as subunit of TRAPP complex in two independent proteomic studies^14^,^15^. It has no ortholog in yeast. It was suggested that TRAPPC12 was a subunit of TRAPPIII. TRAPPC12 and TECPR1 acted at distinct steps in autophagy and depletion of TRAPPC12 and/or TECPR1 increased in the number of autophagosomes and increase autophagic flux^15^. Subsequent studies found that depletion of TRAPPC12 resulted in Golgi fragmentation and blocked trafficking of ts045-VSV-G-GFP. Recently TRAPPC12 per se was shown to have moonlighting function during mitosis by regulating kinetochore stability and CENP-E recruitment, and therefore was renamed TRAMM^16^.

The interaction between TRAPP and COPII vesicle is likely initiated at the ERES in mammalian cells^17^, and is more extensive than Bet3p/TRAPPC3-Sec23^5^,^18^. TRAPPC2 was reported to promote Sar1 dissociation from membrane in order to allow transport of such large protein as procollagen II^19^. Furthermore, COPII coat subunits were identified in mass spectrometry analysis of immunoprecipitates of TRAPPIII complex^20^. Precisely how TRAPPIII may functions to regulate COPII vesicle was far from elucidation. In this study, we uncovered an interaction between TRAPPIII specific subunit TRAPPC12 and COPII specific subunit Sec13/Sec31 tetramer, and this interaction positively modulated the assembly of Sec13/Sec31 tetramer onto COPII vesicle.

## Results

### TRAPPC 12 was a TRAPPIII specific subunit together with TRAPPC8

To determine the function of TRAPPIII in mammalian cells, we developed an antibody that specifically recognized TRAPPC12. This antibody, after affinity purification, was capable of immunoblotting, immunoprecipitation and immunofluorescence staining. Immunoblotting of lysates from several cell lines revealed that the antibody recognized specific bands varying from 90 kD among the cell lines (asterisks, left panel, Supplementary Figure 1A). TRAPPC12 had 10 transcript variants with a large range of protein sizes. The detected bands likely represented tissue-specific expression of certain TRAPPC12 transcript in the cell lines we investigated. Of note, the full-length rodent TRAPPC12 was approximately 50 amino acids larger than human ortholog, and therefore, TRAPPC12 was detected to be almost 100 kD in CHO-K1 cells, in which its protein expression was the strongest among the cell lines tested. Furthermore, when the antibody was first pre-adsorbed with TRAPPC12 antigen, it no longer could detect the indicated bands by immunoblotting (right panel, Supplementary Figure 1A). Pre-immune serum of the same rabbit did not detect any protein bands in the cell lysates (Supplementary Figure 1B). Of the cell lines investigated, α-TRAPPC12 antibody picked up the most non-specific signals from HeLa cell lysate. However, when we performed immunoprecipitation using the antibody, only the 90 kD TRAPPC12 protein was enriched in the immunoprecipitate (Supplementary Figure 1C). This protein was not present in cell lines that were deleted with TRAPPC12 gene (Supplementary Figure 1C and later in the result section). Antigen pre-adsorption also reduced the immunofluorescence signal of α-TRAPPC12 in CHO cells (Supplementary Figure 1D). In HeLa cells deleted with TRAPPC12 gene, TRAPPC12 antibody detected very low signal (Supplementary Figure 1E), suggesting the largely cytosolic TRAPPC12 signal in wildtype HeLa cells were specific. Together, the TRAPPC12 antibody was of good quality for our study of TRAPPC12 functions.

**Figure 1:**
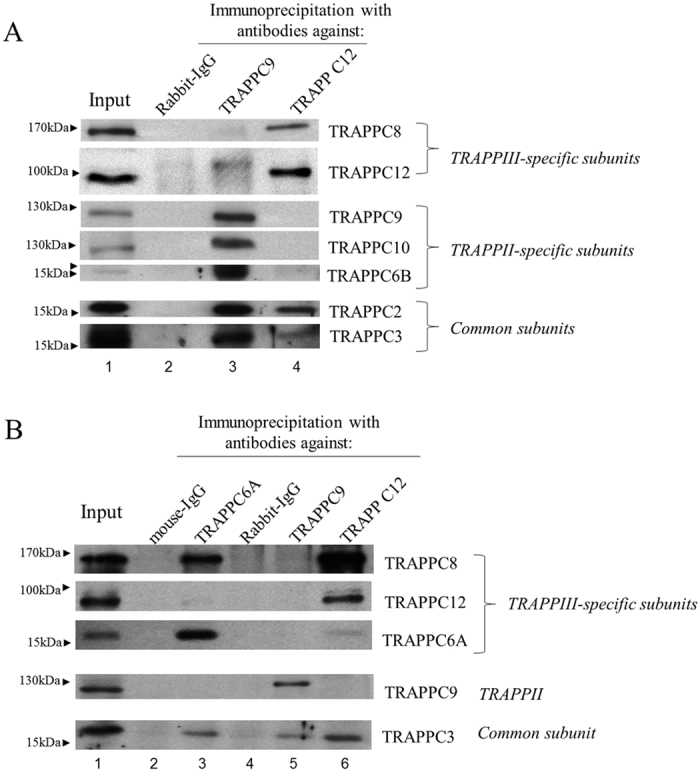
TRAPPC12 and TRAPPC8 were TRAPPIII-specific subunits. (**A**) HeLa cell lysates were subjected to immunoprecipitation using antibody specific to TRAPPC9 and TRAPPC12 and rabbit-IgG as negative control. The different subunits of TRAPP complexes in the immunoprecipitates were detected by immunoblotting using the indicated antibodies specific to these proteins. (**B**) A comparison of subunit composition between TRAPPC6A IP, and TRAPPC9 and TRAPPC12 IP’s. Immunoprecipitations with mouse monoclonal TRAPPC6A antibody was performed and mouse IgG was used as negative control. IP’s with TRAPPC9 and TRAPPC12 were similar to those shown in (**A**). In both panels, approximately 1% of the input lysates and 20% of the immunoprecipitates were loaded onto the SDS-PAGE.

We previously developed an antibody against TRAPPC9^21^, a TRAPPII specific subunit. Using these two antibodies, we unequivocally demonstrated the existence of TRAPPII and TRAPPIII in mammalian cells by immunoprecipitation experiment (Fig. 1). It was clear that TRAPPIII contained the subunits TRAPPC8 and TRAPPC12, whereas TRAPPII contained TRAPPC9, TRAPPC10 and TRAPPC6B (Fig. 1A). The antibody against TRAPPC6A happened to be able to perform immunoprecipitation, and therefore, we investigated what other TRAPP subunits were co-isolated by immunoprecipitation using this antibody (Fig. 1B). TRAPPC8, and to a lesser extent, TRAPPC12 was co-purified in the TRAPPC6A precipitation. This result established that TRAPPC6A was a TRAPPIII-specific subunit whereas TRAPPC6B was TRAPPII-specific, although we did observe trace amount of TRAPPC6B in TRAPPIII precipitation (Fig. 1A and data not shown). Nonetheless, we were certain that TRAPPC12 was a specific subunit of TRAPPIII.

### TRAPPC12 was localized to the ER exit sites and bound to Sec13/31 tetramer

**Figure 2:**
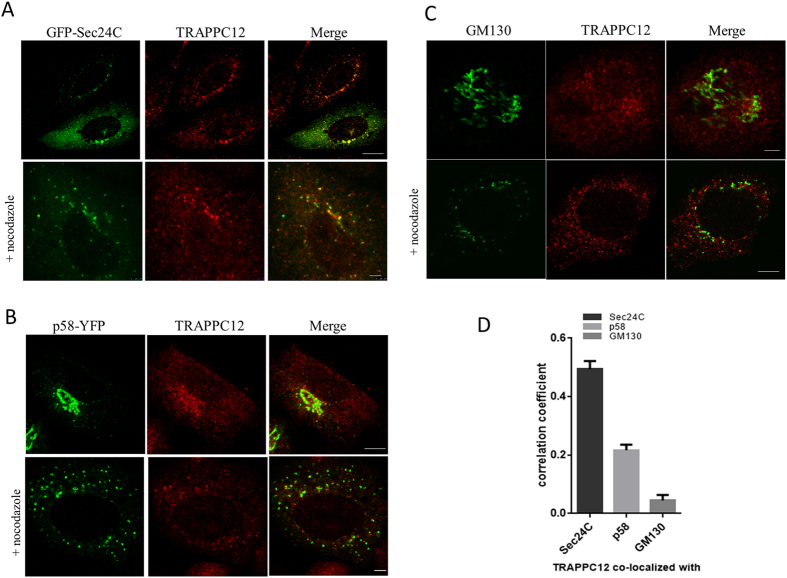
Immunofluorescence colocalization studies on TRAPPC12. Colocalization of TRAPPC12 with various markers by immunofluorescence. (**A**) TRAPPC12 was colocalized with ER exit site marker GFP-Sec24C; (**B**) TRAPPC12 was colocalized with ERGIC marker p58-YFP. (**C**) TRAPPC12 was colocalized with Golgi markers GM130. TRAPPC12 signal was detected by incubating the cell samples purified antibody against TRAPPC12 followed by AF568-conjugated secondary antibodies (**A**,**C**), or AF635-conjugated secondary antibodies (**B**). ER exit sites and ERGIC were detected by the fluorescence signals of transfected GFP-Sec24C and p58-YFP, respectively (**A**,**B**). Golgi was detected by staining the samples with monoclonal antibody against GM130, followed by AF488-conjugated secondary antibody (**C**). In samples where nocodazole was used, 10 μg/ml of nocodazole was added to the cells for 1 hour before fixation and staining. Scale bar is 10 μm (upper) and 2.5 μm (lower). (**D**) Quantitative analysis of TRAPPC12 co-localized with different organelle markers in nocodazole-treated cells. Pearson’s correlation coefficients were 0.49 (TRAPPC12 & Sec24C), 0.22 (TRAPPC12 & p58) and 0.04 (TRAPPC12 & GM130). The coefficients were generated using ImageJ software and specific plugins. The data were shown Mean ± S.D., n > 3.

Immunofluorescence staining of TRAPPC12 revealed a variety of subcellular localizations. Because CHO-K1 cells had the strongest TRAPPC12 protein expression, we performed localization studies in this cell line. TRAPPC12 was in cytosol and in punctate structures that were well colocalized with GFP-Sec24C, p58-YFP and GM130 (top panels, Fig. 2A–C). Sec24 is a subunit of the COPII vesicle coat and a marker for the ERES in mammalian cells, and p58-YFP was a marker for the ER-Golgi intermediate compartments, whereas GM130 is a marker for cis-Golgi. Treatment with nocodazole depolymerized microtubule and broke up the membrane compartments in the secretory pathway into small fluorescent puncta. TRAPPC12 signals were mostly colocalized with GFP-Sec24C and p58-YFP but poorly with GM130 (bottom panels, Fig. 2A–C). The status of how TRAPPC12 was colocalized with these markers upon nocodazole treatment was quantified using the Pearson’s correlation coefficient (Fig. 2D). This analysis suggested TRAPPC12 and GFP-Sec24C were best colocalized (Pearson’s coefficient = 0.49), followed by p58 (0.22), and least with GM130 (0.04). In HeLa and HEK293T cells, TRAPPC12 signals were largely cytosolic and weak punctate staining in proximity of the markers for early secretory pathway was also observed (Supplementary Figure 2A and B). In COS cells and to a lesser degree in HeLa cells, however, TRAPPC12 was also present as a fluorescent puncta in the pericentriolar region of the cells, suggesting a concentration of TRAPPC12 signals in the centrosome and the pericentriolar region. Consistent with this observation, TRAPPC12 was reported to interact with CENP-E and regulate its recruitment onto the kinetochore. Since CHO cells had the strongest protein expression of TRAPPC12 by immunoblotting, we strongly believed that its localization at ERES was physiologically meaningful and that TRAPPC12 likely had functions in the early secretory pathway at the level of ERES, in addition to the reported functions in autophagy and at the centrosome.

### TRAPPC 12 could directly bind to the outer coat complex of COPII vesicle

**Figure 3:**
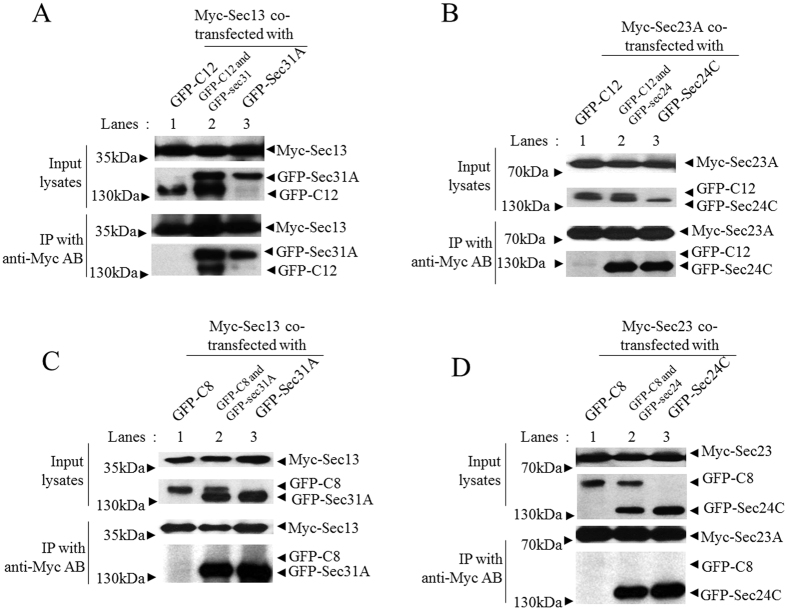
TRAPPC12 directly binds to the outer coat complex of COPII vesicle. GFP-TRAPPC12 or GFP-TRAPPC8 was overexpressed with indicated combinations of plasmids expressing Myc- or GFP-tagged COPII coat subunit(s) in 293T cells. Immunoprecipitations were performed using 9E10 anit-c-Myc antibody and detections of co-precipitated proteins were detected by immunoblottings using the indicated antibodies (anti-c-Myc or anti-GFP). Approximately 1% of the input lysates and 20% of the immunoprecipitates were loaded. (**A**) GFP-TRAPPC12 (GFP-C12) was tested for interaction with Myc-Sec13 alone or with GFP-Sec31A. (**B**) GFP-TRAPPC12 was tested for interaction with Myc-Sec23A alone or with GFP-Sec24C. (**C**) GFP-TRAPPC8 (GFP-C8) was tested for interaction with Myc-Sec13 alone or with GFP-Sec31A. (**D**) GFP-TRAPPC8 was tested for interaction with Myc-Sec23A alone or with GFP-Sec24C.

Based on the immunofluorescence localization study and our previous mass spectrometry identification of Sec13 as an interacting protein of TRAPPC12 (data not shown), we tested if TRAPPC12 interacted with COPII subunits. We co-overexpressed Sec13 and/or Sec31A with TRAPPC12, and then performed co-IP experiments. Sec13 and Sec31A were outer-layer subunits of the COPII coat normally exist as heterotetramers^18^. We found that TRAPPC12 could bind to Sec13 minimally if any, and the binding increased drastically when Sec31 was also co-expressed (Fig. 3A). In comparison, co-expression of Sec23 and Sec24 could not co-precipitate TRAPPC12 (Fig. 3B). Similarly, little if any TRAPPC8 was co-precipitated by COPII coat subunits (Fig. 3C,D). Immunoprecipitation of TRAPPC12 also brought down both Sec31A and Sec13 substantially, but such interaction was non-existent when either Sec protein was tested individually (data not shown). From these experiments, we concluded that TRAPPC12 interacted with tetrameric Sec13/Sec31A.

### Loss of TRAPPIII function reduced recruitment of Sec13/Sec31 onto COPII vesicles

**Figure 4:**
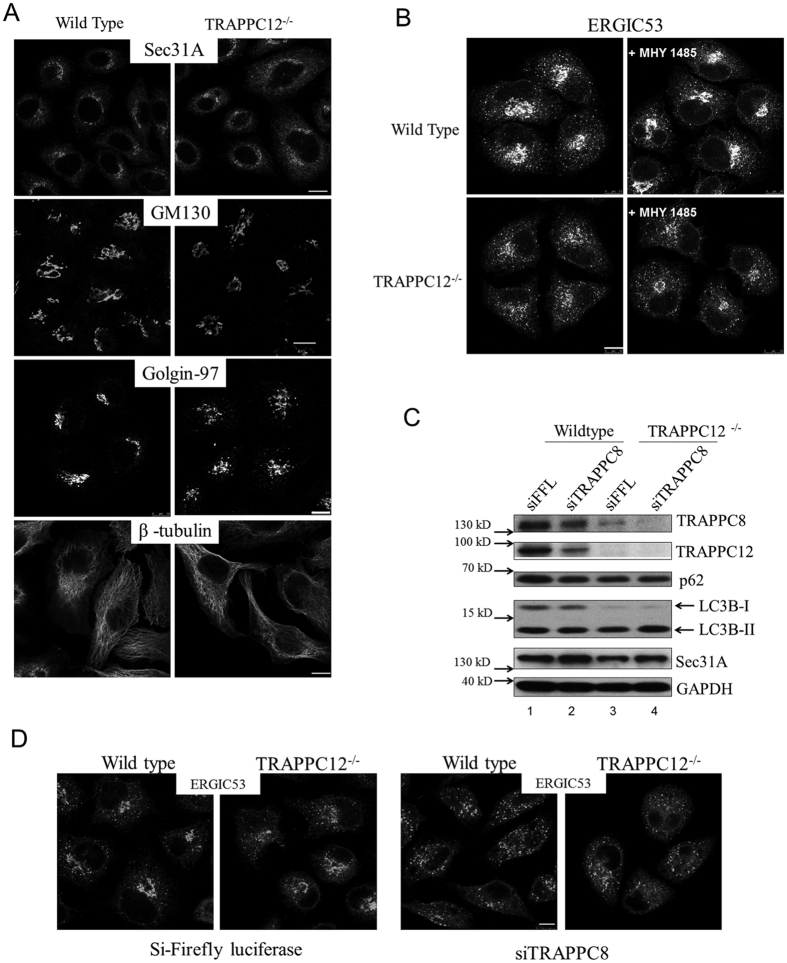
Analysis of the early secretory pathway in TRAPPC12^−/−^ cells. (**A**) Wildtype and TRAPPC12^−/−^ HeLa cells were investigated with indicated markers of the early secretory pathway. (**B**) Wildtype and TRAPPC12^−/−^ HeLa cells were stained with ERGIC53 after the cells were treated with or without autophagy inhibitor MHY 1485 at 10 μM for 2 hours. (**C**) Lysates of wildtype and TRAPPC12^−/−^ HeLa cells were immunoblotted to detect the indicated proteins. (**D**) Wildtype and TRAPPC12^−/−^ HeLa cells were depleted with siRNA specific to Firefly luciferase (control) or TRAPPC8 sequence and then stained with antibody against ERGIC-53. Scale bars = 10 μm.

To begin to investigate how the interaction between TRAPPC12 and Sec13/Sec31A complex may regulate COPII vesicle formation, we constructed TRAPPC12 deletion cell lines by CRISPR-Cas9 method. Detailed strategy of genomic deletion, detection and isolation of TRAPPC12 deleted cell clones, and confirmation of TRAPPC12 deletion were described in Supplementary Figure 3. We initially performed deletion in HEK293T cells due to the high transfection efficiency in this cell line. As shown in Supplementary Figure 3A, at least four clones isolated to be homozygous TRAPPC12 deletion. Subsequently, we obtained at least six TRAPPC12 deletion clones from HeLa cells and proceeded with morphological analysis with clones from this cell line because HeLa cells were flat and contained larger volume of cytosol (Supplementary Figure 3E). When the early secretory pathway was investigated, ERES marker Sec31A was slightly more cytosolic in these cells compared to wildtype HeLa (Fig. 4A, top panels). The Golgi was slightly weaker or fragmented as shown by the stainings of Golgi matrix protein GM130 and trans-Golgi marker Golgin-97, respectively (Fig. 4A, second and third panels). Microtubule was not observed to be different (Fig. 4A, bottom panels). The ERGIC in TRAPPC12^−/−^ cells was most drastically dispersed compared to wildtype HeLa cells (Fig. 4B, left panels). As reduced activity of TRAPPC12 and TRAPPC8 both caused increased level of autophagy, we suspected the change at ERGIC (and potentially other compartments such as the Golgi) was due to increased autophagy. Therefore, we incubated the cells with an autophagy inhibitor MHY1485 before we determined the ERGIC morphology. As shown in the right panels of Fig. 4B, MHY1485 slightly caused a concentration of the ERGIC signals around the pericentriolar region of both the wildtype and TRAPPC12 deleted cells, but the weaken and dispersed pattern was not rescued, suggesting the deletion of TRAPPC12 caused a direct effect at the early secretory pathway. siRNA depletion of TRAPPC8 also caused dispersal of the markers in early secretory pathway (Supplementary Figure 4), suggesting TRAPPC8 and TRAPPC12 must function together as TRAPPIII to affect the early secretory pathway, and therefore, inactivating either subunit caused very similar abnormalities in the early secretory pathway. This notion was further supported by the observation that depleting/deleting either subunit induced protein degradation of the other. When we systematically investigated the lysates from TRAPPC12^−/−^ cells and TRAPPC8 siRNA depleted cells by immunoblotting, we found that TRAPPC8 protein level was significantly reduced in TRAPPC12 deleted cell lysate (lanes 1 and 3, top panel, Fig. 4C). Similarly, siRNA depletion of TRAPPC8 reduced protein level of TRAPPC12 (compare lane 1 and lane 2, second panel, Fig. 4C). As expected, LC3-II to LC3-I ratio was increased in TRAPPC12^−/−^ cells but autophagy adaptor protein p62 and Sec31A were not changed (Fig. 4C). We depleted TRAPPC8 in TRAPPC12^−/−^ cells to see if inactivating both TRAPPIII subunits worsened the status of the ERGIC but such treatment only resulted in slightly more dispersal of ERGIC signals (Fig. 4D), suggesting that inactivating either TRAPPC8 or TRAPPC12 already impaired the TRAPPIII function on the early secretory pathway. We further investigated if the spatial relationship between ERGIC and ERES were different in the TRAPPIII defective cells. ERGIC fluorescence marker construct YFP-p58 was transfected into cells deleted and/or depleted with TRAPPC12 and TRAPPC8, and then co-staining with Sec31A was performed (Supplementary Figure 5). Normally ERGIC and ERES signals are juxtaposed and this property was not changed in TRAPPIII deleted/depleted cells. Together with the interaction study (Fig. 3), these results suggested TRAPPIII-Sec13/Sec31A interaction must regulate the COPII vesicles and when TRAPPIII activity was compromised *in vivo*, post-ERES membrane compartments such as ERGIC and Golgi became abnormal.

### TRAPPC12 regulates the membrane binding of COPII coat subunits in live cells

**Figure 5:**
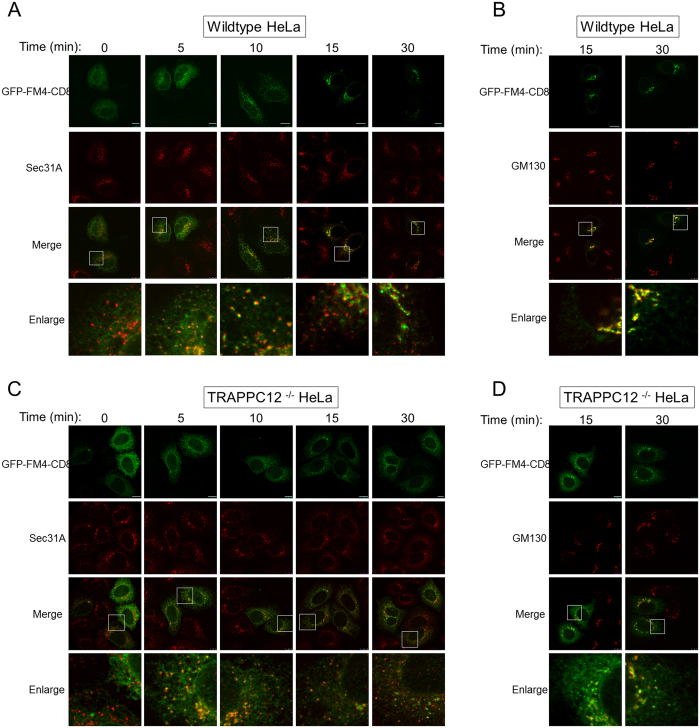
ER-to-Golgi transport in wildtype and TRAPPC12^−/−^ HeLa cells. ER-to-Golgi transport was monitored by the transfected GFP-FM4-CD8 as transport marker. GFP-FM4-CD8 accumulated in the ER lumen in aggregated form until D/D solubilizer was applied to the cells for the indicated time. ER-to-Golgi traffic was monitored by co-staining with ERES marker Sec31A (**A**,**C**), or with Golgi marker GM130 (**B**,**D**). Scale bars = 10 μm.

To begin to determine whether TRAPPIII regulate ER-to-Golgi traffic, we monitored the transport of a fluorescent marker protein GFP-FM4-CD8^22^. This GFP fusion protein aggregated at the ER due to the FM domains. When a solubilizing agent was applied to the transfected cells, the fusion protein could exit the ER and transported to the Golgi and eventually to the plasma membrane. In five to ten minutes after D/D solubilizer was applied, GFP-FM4-CD8 exited the ER and became colocalized with ERES marker Sec31A extensively in wildtype HeLa cells (Fig. 5A). At 15 and 30 minutes, the colocalized signal with Sec31A was reduced, suggesting the cargo protein had moved to the Golgi. This was confirmed by the extensive colocalization with Golgi marker GM130 (Fig. 5B). In TRAPPC12 deleted HeLa, however, the export of GFP-FM4-CD8 from ER appeared to be comparable to wildtype cells in the first 10 minutes (Fig. 5C), but fluorescent signal largely remained in the ERES throughout 30 minutes. Signals colocalized with the Golgi only began to appear at 30 minute time point (Fig. 5D). Overall, this experiment demonstrated that TRAPPC12 deletion caused a traffic delay at the ER-to-Golgi step, consistent with the report that trafficking of VSV-G was blocked by siRNA depletion of TRAPP subunits including TRAPPC8 and TRAPPC12^12^.

**Figure 6:**
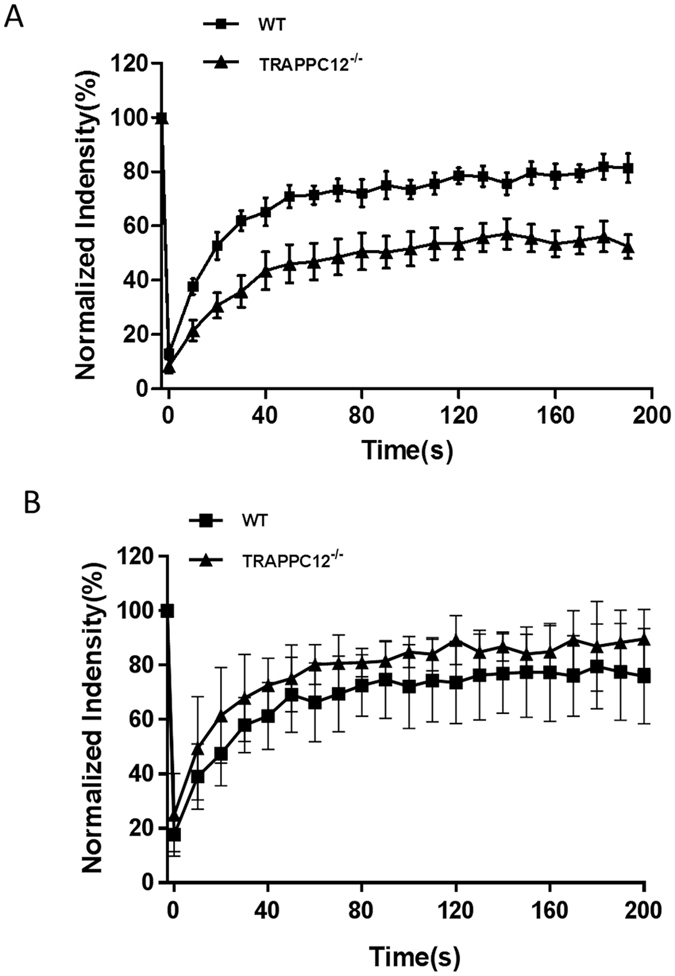
FRAP analysis of GFP-Sec31A wildtype and TRAPPC12^−/−^ HeLa cells. (**A**) Wildtype (square) or TRAPPC12−/− (triangle) HeLa cells transiently transfected with GFP-Sec31A was bleached by laser and the recoveries of GFP fluorescence were recorded. Examples of detailed FRAP operations were documented in Supplementary Figure 5. (**B**) Wildtype (square) or TRAPPC12−/− (triangle) HeLa cells transiently transfected with GFP-Sec24C were bleached by laser and the recoveries of GFP fluorescence were recorded. n = 7; Error bars = S.D.

Because of the interaction between TRAPPIII and COPII outer coat subunits, delay of ER-to-Golgi traffic by TRAPPC12 deletion could be caused by dysregulation of COPII vesicles. To investigate how TRAPPC12 deletion might affect COPII vesicles, we took advantage of fluorescence recovery after photobleaching (FRAP) to determine the rates of recovery of GFP-Sec31A on ER exit site both in wildtype and TRAPPC12^−/−^ cells. Examples of detailed recordings of the fluorescence images were documented in Supplementary Figure 6. From the FRAP data, GFP-Sec31A recycled at an average half-life of recovery of 17.4 seconds in wildtype HeLa cells, versus 22.4 seconds in TRAPPC12^−/−^ cells (Fig. 6A). The amount of immobile fraction was also increased from an average of 21% in wildtype cells to 52% in the TRAPPC12^−/−^ cells. Under the same condition, the recovery rates of GFP-Sec24C was indistinguishable between wild type and TRAPPC12^−/−^ cells (Fig. 6B), suggesting the inner layer of COPII coat was not affected by defective TRAPPIII in these cells. These results suggested that the outer-layer of COPII coat was less capable of engaging in dynamic assembly and disassembly of the COPII vesicle coat formation when the function of TRAPPIII was compromised. As a result, a significant portion of the membrane bound Sec31A turned into immobile fraction in FRAP.

**Figure 7:**
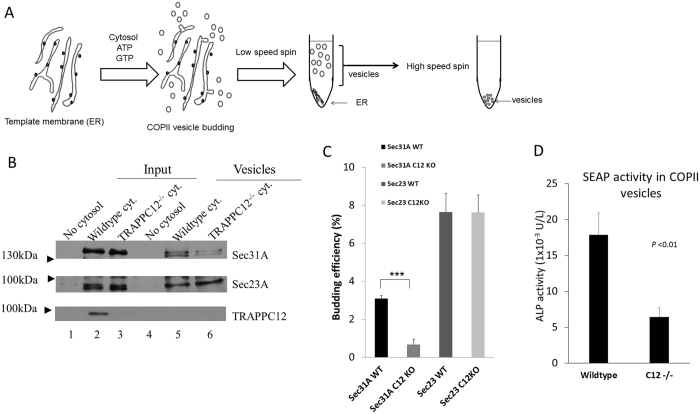
*In vitro* COPII budding reaction supported by wildtype or TRAPPC12^−/−^ cytosol. (**A**) Schematic drawing of the procedures of *in vitro* COPII budding assay. (**B**) A representative experiment of COPII budding reaction. ER membranes from wildtype HeLa cells were mixed with no cytosol (lanes 1 and 4), wildtype cytosol (lanes 2 and 5), or TRAPPC12^−/−^ cytosol (lanes 3 and 6) to allow COPII vesicles budding to occur. 10% input and 100% of budded vesicles were loaded on SDS-PAGE. (**C**) Quantitative analysis of COPII budding assay. Sec31A- and Sec23A-specific bands were quantified by ImageJ software. The budding efficiencies were 3.08% (Sec31 WT), 0.68% (Sec31, C12 KO), 7.64% (Sec23 WT), and 7.62% (Sec23, C12 KO) of the corresponding input signals (i.e., comparing signals of lane 5 to lane 2, and lane 6 to lane 3). The data were shown Mean ± S.D., n = 3, P < 0.01. (**D**) COPII budding reactions were carried using ER template membranes isolated from a HeLa cell line stably transfected with SEAP as transport cargo. The budded COPII vesicles were measured with alkaline phosphatase activity as an indication of cargo transport. The data represented averages of above-background alkaline phosphatase activities of the two samples, using no cytosol control as background. Error bars = S.D., n = 3, P < 0.01.

To confirm the observation that TRAPPIII regulated COPII coat outer layer, we adopted an *in vitro* COPII budding assay developed by Kim *et al*.^23^,^24^. In this experiment, we isolated ER enriched membrane fractions as template and mixed with cytosols that were independently isolated and allowed vesicles to be budded in an *in vitro* condition. Then the vesicles were separated from the template membranes by density gradient centrifugation. A schematic drawing of how this assay was performed is shown in Fig. 7A. From this experiment, we found that COPII vesicles budded from cytosol of TRAPPC12^−/−^ cells contained less Sec31A protein than those budded form wildtype cytosol, while vesicle-bound Sec23A was similar between two groups (compare lane 5 and 6, Fig. 7B). Quantification of three independent experiments showed that the amount of Sec31A on COPII vesicles was found to be approximately 3.1% of total input cytosol from wildtype HeLa cells, versus 0.68% from TRAPPC12 deleted cells (Fig. 7C). Of note, although TRAPPC12 clearly played a role in modulating the level of Sec31A on budded COPII vesicles, TRAPPC12 protein was not detected on the budded vesicles (bottom panel, Fig. 7). Furthermore, the reduction of Sec31A strictly correlated with the TRAPPC12^−/−^ cytosol applied to the budding reaction. ER membranes from TRAPPC12^−/−^ cells did not generate any change of Sec31A level on the budded vesicles (data not shown). Overall, this experiment demonstrated biochemically that the COPII outer-layer was, indeed, less efficient in being assembled onto COPII vesicles.

To determine if the COPII vesicles produced with the support of TRAPPC12^−/−^ cytosol actually reduced transport of cargo protein, we took ER membranes from a cell line that was stably transfected with a secreted form of alkaline phosphatase (SEAP)^25^,^26^, and determined the amount of alkaline phosphatase (AP) activity from the budded COPII vesicles. As shown in Fig. 7D, the COPII vesicles produced with wildtype cytosol contained approximately 18 mU/L AP activity, whereas those produced with cytosol deleted with TRAPPC12, however, contained less than 6.5 mU/L AP activity. These activities corresponded to approximately 1.7% and 0.6% of the AP activity contained in the input ER membrane template, respectively.

## Discussion

In the present study, we found that TRAPPC12 was located in the cytosol and on ERES. TRAPPC12 acted as a positive modulator of the recruitment of outer layer of COPII coat, Sec13/Sec31, onto COPII vesicles. Without functional TRAPPIII, a slow-down in the dynamic assembly of the outer layer coat in COPII vesicle formation was observed both in the TRAPPC12^−/−^ cells. Increased portion of Sec31A onto ERES turned into immobile fractions and this shift in FRAP explains why there was little change in the fluorescence signal we observed of Sec31A at ERES in TRAPPC12^−/−^ cells (Fig. 4A). To our surprise, reduced alkaline phosphatase (cargo) was observed in *in vitro* COPII budding reaction conducted with the support of TRAPPC12^−/−^ cytosol. The COPII vesicles produced contained less outer-layer COPII subunits but the amount of inner-layer subunits was comparable to wildtype control. Outer-layer subunits were not known to directly participate in cargo selection or concentration, with the exception of large-sized cargo, procollagen II^19^. Although how faithfully the *in vitro* budding assay recapitulates COPII vesicle production *in vivo* remains a subject of debate, our *in vitro* COPII budding result indicates that TRAPPIII and/or the outer layer of COPII coat may also contribute to cargo selection or concentration during COPII budding at least in *in vitro* setting. This notion seems plausible in view of the reported function of TRAPPC2 on COPII vesicles.

TRAPPC2 was reported to modulate COPII vesicles in manner similar to the functions of Tango1 and ALG-2^27^,^28^. siRNA depletion of TRAPPC2 caused a block of ER export of a large cargo protein pro-collagen II while smaller transport cargo protein such as the VSV-G was not affected. TRAPPC2 was required to modify the lattice of the COPII coat by changing the rate of GTP hydrolysis of Sar1, so that the vesicle was enlarged to accommodate pro-collagen II^19^. Although both TRAPPC2 and TRAPPC12 targets the outer layer of COPII, we believe the finding reported in this study is distinct from those observed of TRAPPC2 because ER export of small cargo proteins was delayed in TRAPPC12 deleted cells. When we monitored the traffic of GFP-VSV-G-tsO45 (data not shown) and GFP-FM4-CD8 (Fig. 5), both of which were readily accommodated into typically sized COPII vesicles, we found longer time was required for the cargos to move from ERES to the Golgi. This finding was consistent with those reported by Scrivens *et al*., in which siRNA depletion of TRAPPC12 caused a block in ER-to-Golgi traffic for VSV-G. A severe block at this step usually causes redistribution of Golgi enzymes and Golgins to the ER and ultimately affects the survival of the cells. Since TRAPPC12 deleted cells are viable, and the status of their Golgi and ERGIC are mildly affected, we believe TRAPPIII is limited to a modulating role, rather than an essential one in ER-to-Golgi traffic.

Yeast TRAPPI serves as a COPII vesicle tether. Evidence for TRAPP as COPII vesicle tether has been reported. Common subunit TRAPPC3 (a.k.a mBet3) is localized to ERES and neutralizing antibody specific to this subunit blocked ER-to-Golgi traffic *in vivo* and homotypic COPII vesicle tethering *in vitro*^17^, suggesting the function of TRAPPI was inhibited. Both TRAPPC3 and TRAPPC2 (a.k.a. sedlin) interact with the inner layer of COPII coat. Depletion of TRAPPC2 blocked ER export of megacarrier Pro-collagen II but not smaller carrier such as VSVG^19^. However, when TRAPPC2 was more efficiently depleted, Golgi also became fragmented^19^, implicating general secretion at the step of ER-to-Golgi was affected. As TRAPPIII is also ERES-localized and interacts with COPII outer layer, the finding reported here will suggest COPII vesicles interact with two forms of TRAPP (i.e. TRAPPI and TRAPPIII), a rather redundant mechanism. Therefore, it is possible that TRAPPIII is, in fact, the COPII tether in mammals. Yet, there are evidence implicating TRAPPIII is distinct from the putative TRAPPI in mammalian cells. All forms of TRAPP arguably serve as GEF for Ypt1 in yeast. In mammals, although TRAPPI has not been clearly demonstrated, TRAPPII and TRAPPIII can be detected by immunoprecipitation (Fig. 1). When these complexes were tested for their abilities to stimulate guanine nucleotide exchange for Rab1, only TRAPPII complex contained Rab1 GEF activity. Rab1 is essential to ER-to-Golgi traffic, but ER-to-Golgi traffic is normal in cells deleted with TRAPPII specific subunits, and the localization of Rab1 is also normal in these cells (data not shown). As TRAPPII is not responsible for activating Rab1 *in vivo* and TRAPPIII cannot activate Rab1 in mammalian cells, the only logical deduction is TRAPPI must exist to regulating Rab1 activity. Our present study reinforces the idea that TRAPPI is responsible for COPII vesicle tethering, while TRAPPIII have modulating effect. Their exact relationship, for example, whether or not TRAPPI can be converted to TRAPPIII during COPII vesicle coat assembly, will require further clarification.

## Materials and Methods

### Cell lines and media

HeLa, HEK293T and COS-7 cells, originally obtained from ATCC (Manassas, VA), were cultured in Dulbecco’s Modified Eagle’s Medium (DMEM, Invitrogen, Cat. No.: 12800-017) supplemented with 10% fetal bovine serum (FBS, Sigma, Cat. No.: A6003) at 37 °C in 5% CO_2_. CHO-K1 cells were cultured in DMEM supplemented with 10% FBS 0.35 mM L-proline (Sigma, Cat. No. P5607), and 10% fetal bovine serum at 37 °C in 5% CO_2_. Cells were passaged after being detached with Trypsin (Invitrogen, Cat. No. 12604-021) when the confluency reached greater than 90%. When used in experiments, the cells were typically harvested in mid-log growth phase.

### Plasmids

cDNAs encoding mammalian TRAPP and COPII vesicle (Myc-Sec31A, Myc-Sec13, Myc-Sec24C, Myc-sec23A and Myc-sec24D) subunits were amplified by PCR and subcloned into pCMV-Myc (Clontech). GFP-tagged above subunits were constructed by transferring the corresponding cDNA sequences into peGFP-Cx vector (Clontech).

### Antibodies

Rabbit polyclonal antibody against TRAPPC8 and TRAPPC9 were raised as previously described^13^,^21^. Rabbit polyclonal antibody against TRAPPC12 was raised by repeated injection of rabbits with bacterially expressed and purified recombinant TRAPPC12 protein. The anti-sera were subjected to affinity purification using a column coupled with TRAPPC12 protein. Detailed procedures of how recombinant TRAPPC12 was coupled onto the resin followed manufacturer’s instruction (Aminolink^®^ plus coupling resin, ThermoFisher Scientific, Cat no. 20501). Briefly, 3–5 ml of TRAPPC12 anti-serum was subjected to affinity purification in a column containing approximately 1 mg of TRAPPC12 protein. After extensive washes with 1X PBS, the antibody was eluted with 1 ml fractions of 0.1 M glycine, p.H. 2.4. The antibody containing fractions were neutralized with approximately 0.1 ml of Tris, p.H. 8.8. The eluted antibody was concentrated and stored at −80 °C. Experiments involved in generation of antibodies from rabbits were performed in accordance with relevant guidelines and regulations. The relevant protocols were approved by the Animal Experimentation Ethics Committee, Faculty of Medicine, The Chinese University of Hong Kong.

Rabbit polyclonal antibody against GFP (sc-8334), mouse monoclonal antibodies against c-Myc, 9E10 (sc-40) and Mouse monoclonal antibody against TRAPPC6A(G-5, sc-376032) were purchased from Santa Cruz Biotechnology, Inc. (CA, USA). Monoclonal antibody against Golgin-97(Invitrogen) was previously obtained from Dr. Wing Keung Liu’s laboratory (The Chinese University of Hong Kong). Monoclonal antibody against Sec31A (612350), and against GM130 (610823) were purchased from BD Biosciences. Monoclonal antibody against ERGIC-53 was a gift from Hans-Peter Hauri (University of Basel, Switzerland). Alexa Fluor (AF) conjugated secondary antibodies [AF488 goat anti-mouse (A11017), AF568 goat anti-mouse (A11019), AF635 goat anti-mouse (31575), AF488 goat anti-rabbit (A11070) and AF568 goat anti-rabbit (A21069)] were purchased from Invitrogen. Horseradish Peroxidase Conjugated secondary antibodies were purchased from Rockland.

### Transfection

Polyethylenimine (PEI, Sigma, Cat. No. 408727) was used as transfection reagent for the cell lines described in this study. Cells were cultured to 70–80% confluency before transfection. Typical amount of DNA plasmid is 0.4 μg in 4-well plate (diameter is 14 mm) for immunofluorescence and 10 μg in 10-cm culture dish for immunoprecipitation. Take 1 × 10 cm dish as example, 10 μg of plasmid was diluted in 400 μl of 1 × PBS and 25 μg of PEI was diluted into 400 μl of 1 × PBS separately, mixed by vortex and stayed at room temperature for about 5 minutes. The two parts were mixed together by vortex and stayed at room temperature for about 15 minutes. Then, the mixture was dropwisely added to the culture dish with swirl and incubated for four hours before changing the culture medium to normal growth medium. Transfections for 4 well plate were carried out with 1 μg of PEI.

### Immunoprecipitation

After transfecting with the desired plasmids for at least 18 hours, the cells in 10-cm plates were washed with 1 × PBS for 3 times, lysed in 1 ml lysis buffer (20 mM Tris, pH 8.0, 100 mM NaCl, 0.1% NP-40) plus 1 × protease inhibitor cocktail Complete H (Roche, Cat. No.: 3RA04693132001), and scraped and collected into 1.5 ml tubes. The lysates were centrifuged at 10,000 rpm for 10 minutes at 4 °C. The supernatants were subjected to immunoprecipitation. 50 μl of lysate would be taken and reserved as input in subsequent analysis. Protein-A sepharose slurry (sigma, Cat. No.: P3391) and the required primary antibody (9E10 anti-c-Myc) were added to the lysates and incubated. Incubation was carried out for more than 3 hour with agitation at 4 °C. Then the immunoprecipitates were washed with lysis buffer for 3 times. The samples were prepared for SDS-PAGE gel and immunoblotting analysis after lysis with 2 × SDS-PAGE sample buffer. For immunoprecipitation of native TRAPPII and TRAPPIII complex, HEK293T cells from 15-cm plate were harvested and lysed as described above. The lysates were subjected to immunoprecipitation using 2 μg of antibodies specific for TRAPPC9 and TRAPPC12, respectively.

### CRSPR/Cas9 induced deletion of TRAPPC12

Three pairs of RNA sequences targeting to human TRAPPC12 gene were designed for TRAPPC12 specific genomic locus. The intended deletion would encompass about 500 base pairs. LentiCRISPR v2 (Addgene plasmid: #52961) was used for the construction of single guide RNA^29^. The sequences are shown below:

C12-1. Forward: 5′-caccGCAGCGCGGCGCCGTGTTCG-3′

Reverse: 5′-aaacCGAACACGGCGCCGCGCTGC-3′

C12-2. Forward: 5′-caccgCCCTCAGCTCGCGCCTCCGG -3′

Reverse: 5′-aaacCCGGAGGCGCGAGCTGAGGGc-3′

C12-3. Forward: 5′-caccgTACTGGGTGCCGCGTCCTCG -3′

Reverse: 5′-aaacCGAGGACGCGGCACCCAGTAc-3′

Different combinations of equal molar sgRNAs of TRAPPC12(Combination 1: 1 + 2; Combination 2: 1 + 3; Combination 3: 2 + 3; Combination 4: all three) were transfected into HEK293T and HeLa cells separately. After 48 hours, fresh medium containing Puromycin at 1~2 μg/ml was added for selection of stable transfectants. After about one week, the Puromycin-resistent cells were collected for transfection efficiency test and the remaining were continued in culture. The groups of cells with higher efficiency of deletion were trypsinized seeded into 96 well plates at dilution optimal for getting single cell clones: About 30~50 cells were diluted into 10 ml medium and then seeded into a 96-well plate with 100 μl per well. After about 2 weeks, each of the single cell clones were expanded and collected for further expansion and for genotyping PCR to identify bona fide knockout clones. Genomic DNA was extracted from these clones for genotyping PCR. The positive genomic PCR products plus scramble control were sent for sequencing in order to confirm the presence of indels in the intended TRAPPC12 locus. Furthermore, the expressions of TRAPPC12 protein from the cell lysates of candidate knockout clones were investigated as additional step for confirming the deletion.

### Fluorescence microscopy

Cells were seeded on 12-mm coverslips for more than 24 hour until they reached to 50% to 70% confluency. The cells were fixed with 3.7% formaldehyde for 15 minutes after being washed several times with 1 × PBS. Then the cells were permeabilized with 0.1% Triton X-100 diluted in 1 × PBS for 5 minutes. Cold absolute methanol fixation was used instead for detection of Golgin-97. PBS plus 1% BSA was added to the cells for blocking for 15 minutes. Unless otherwise indicated, the antibodies were diluted in the blocking buffer with concentration at 1 μg/ml. The primary antibody was incubated for 1 hour at room temperature. After being washed with blocking buffer for 3 times, the sample was incubated with Alexa Fluor conjugated secondary antibody for 30 minutes at room temperature. The cells were mounted on glass slide in Fluoromount-G (Southern BioTech, USA, Cat. No.: 0100-01) after being washed three times in blocking buffer. Images of stained cells were acquired with Leica SP5 laser confocal microscope which was equipped with 63 × water-immersion objective lens. Images were processed with Adobe Photoshop CS6.

Transport of GFP-FM4-CD8 from ER to Golgi was carried out by first transfecting the indicated cells with GFP-FM4-CD8. The next day, the cells were applied with D/D solubilizer (Clontech, cat. no. 635054) at 0.5 μM for the indicated amount of time. Then, the samples were fixed and stained with Sec31A or GM130 before visualization in confocal microscope.

### Time-lapse and Fluorescence recovery after photobleaching (FRAP) analysis

Wild type HeLa cells (WT) and TRAPPC12^−/−^ cells were cultured into glass bottom cell culture dish (NEST. Cat. No.: 801002Φ15 mm). Prior to FRAP experiments, the cells were transfected with GFP-Sec24C or GFP-Sec31A constructs by ScreenFect™ A (InCella Cat. No: S-3001). 24 hours after transfection, the culture dishes were changed with fresh DMEM medium plus 10% FBS and placed into incubating chamber on the microscope stage. The stage was heated at 37 °C and cells were imaged for less than 3 hours. Time-lapse imaging of live cells was carried in an Olympus FV1200 laser-scanning confocal microscope which was equipped with a 60×, 1.2 numerical aperture (NA) water-immersion objective (UPLSAPO60XW) and a stage incubator system. The time-lapse images were captured before and after bleaching of the GFP signals using a SIM scanner in round scan mode with the 473 nm line of the argon laser set at 50% transmission for 200 milliseconds to bleach the targets. GFP signal was excited with 473 nm line of an argon laser with 5% transmission, and the emission was collected with 520 nm. An optical zoom of 2.2 was used and images were acquired with 10 second interval at a 140 × 140 resolution. A single image of the whole cell was saved as 512 × 512 resolution. For quantitative analysis, the fluorescence intensities in the regions of interest (ROI) and background (cell-free area and cell without bleached) were measured by the FLUOVIEW FV1000 4.2.1.20 software. 11 dots from more than 9 different cells were bleached and monitored in every group.

### *In vitro* COPII budding assay

The COPII budding was modified from the procedure developed by Kim *et al*.^23^,^24^. Briefly, donor membrane and cytosol were separately isolated from HeLa wild type (WT) and TRAPPC12 knockout cells (TRAPPC12^−/−^). Three 10-cm plates of cells with more than 90% confluency were extracted for donor membranes. Ten 15-cm plates of cells with more than 90% confluency were extracted for cytosol. Donor membranes in XX unit of OD600 and cytosol (10 μg) were mixed with10 μl of 10 × ATP regeneration system and B88-0 buffer in a reaction with final volume of 100 μl. The reaction was incubated at 30 °C for 45 minutes. The donor membranes were pelleted by centrifugation for 10 minutes at 10,000 rpm (9,800 g) at 4 °C. 75 μl of the supernatant was transferred to ultracentrifuge tube and spin at 162,000 × g in a HITACHI S140AT-2067 rotor (55,000 rpm) for 20 minutes at 4 °C. The pellet was re-suspended with 10 μl of 2 × WB buffer. The budded COPII vesicles were detected by immunoblotting of COPII coat subunits.

To determine COPII vesicles produced from the *in vitro* budding assay contained transport cargoes, ER template membranes that contained secreted alkaline phosphatase (SEAP) were used. HeLa cells stably transfected with SEAP was obtained from Ayano Satoh^26^. ER template membranes from this cell line were isolated as described above. COPII budding assay was performed in triplicates and the amount of alkaline phosphatase activity was determined by alkaline phosphatase colorimetric assay kit (GeneTex). The activity assay was calibrated with the known standard of alkaline phosphatase enzyme.

## Additional Information

**How to cite this article:** Zhao, S. *et al*. Mammalian TRAPPIII Complex positively modulates the recruitment of Sec13/31 onto COPII vesicles. *Sci. Rep.* **7**, 43207; doi: 10.1038/srep43207 (2017).

**Publisher's note:** Springer Nature remains neutral with regard to jurisdictional claims in published maps and institutional affiliations.

## Supplementary Material

Supplementary Figures
