# Supplementary material for: Mammalian TRAPPIII Complex positively modulates the recruitment of Sec13/31 onto COPII vesicles
Source: Sci Rep. 2017 Feb 27;7:43207. doi: 10.1038/srep43207 (PMC5327430; doi:10.1038/srep43207)
Supplement: Supplementary Figures [file srep43207-s1.docx]

Mammalian TRAPPIII Complex positively modulates the recruitment of Sec13/31 onto COPII vesicles

Shan Zhao, Chun Man Li, Xiao Min Luo, Gavin Ka Yu Siu, Wen Jia Gan, Lin Zhang, William KK Wu, Hsiao Chang Chan and Sidney Yu

**Supplementary information**

**
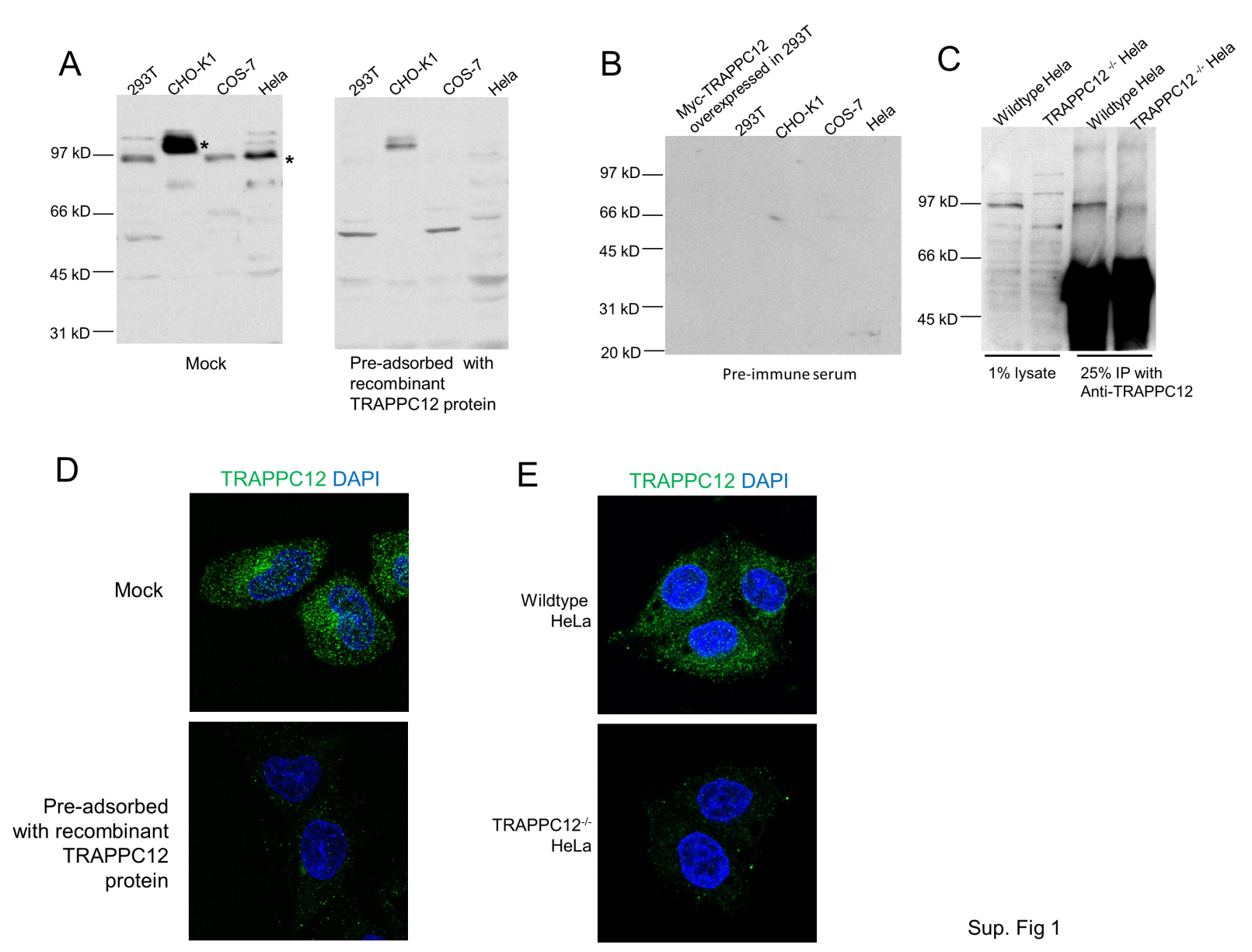
**

Supplementary Figure 1. Characterization of anti-TRAPPC12 antibody

(A) 20 ug of lysates derived from the indicated cells were run on SDS-PAGE and then immunoblotting using the anti-TRAPPC12 antibody was performed. Nitrocellulose strip containing recombinant TRAPPC12 protein was pre-incubated with anti-TRAPPC12 before the antibody was applied to immunoblotting on the cell lysates (right panel). Nitrocellulose strip containing no protein was used in the mock control (left panel). Protein bands with size in 90 kD (or 105 kD for CHO-K1 cells) are likely specific band representing endogenous TRAPPC12 protein (asterisks, left panel).

(B) Pre-immune serum of the same rabbit did not pick up any non-specific protein bands.

(C) TRAPPC12 antibody immunoprecipitated a specific band at approximately 90 kD from cytosol of wildtype HeLa cells but not from cytosol of TRAPPC12 deleted cells. Other non-specific bands were not enriched in the immunoprecipitate.

(D). Anti-TRAPPC12 antibody pre-adsorbed with nitrocellulose strip containing TRAPPC12 protein stained with greatly reduced immunofluorescence signals in CHO cells. Mock treated antibody could stain endogenous TRAPPC12 around the perinuclear region.

(E) Anti-TRAPPC12 antibody stained largely cytosolic and puncta structures in wildtype HeLa cells but greatly reduced fluorescence signals was observed when staining TRAPPC12 deleted HeLa.


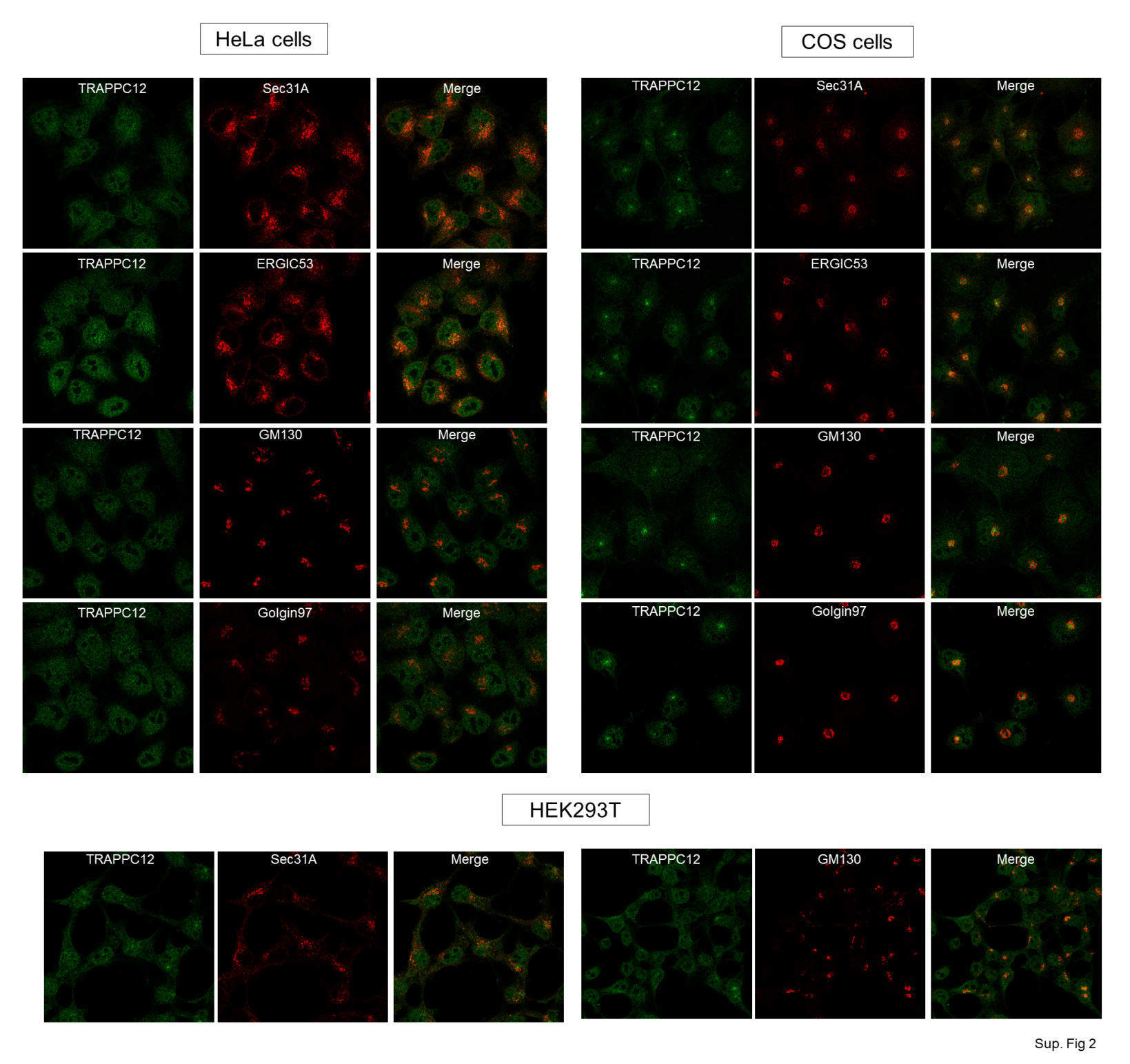


Supplementary Figure 2. Immunofluorescence staining of TRAPPC12 (green) and colocalization with various indicated organelle markers (red) in HeLa, COS and HEK293T cells.


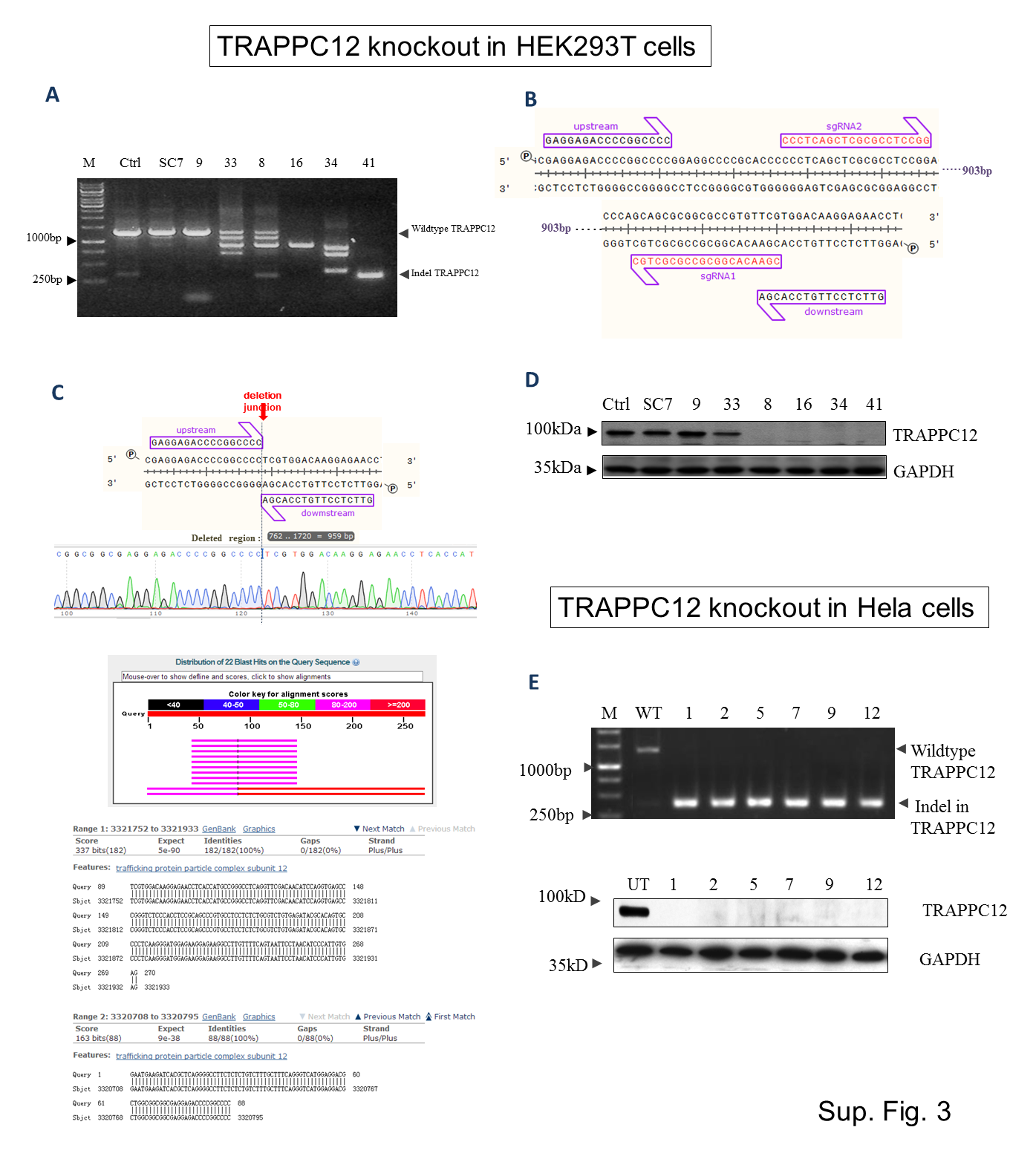


Supplementary Figure 3. Targeted deletion of TRAPPC12 with CRISPR/Cas9 in HEK293T and HeLa cells. (A) Examples of genomic PCR detections of TRAPPC12 knockout clones in HEK293T cells. Genomic DNA from control cells (Ctrl), cells transfected with scrambled sgRNA (SC7), and several candidate TRAPPC12 deleted cell clones were amplified with primers flanking the intended indel locus. From this experiment, clone 9 was likely wildtype, whereas clones 33 and 8 were likely heterozygous for TRAPPC12 deletion. Clones 16, 34 and 41 were likely homozygous deletion for TRAPPC12. (B) Sequence information of TRAPPC12 genomic locus, sgRNA sequences and flanking PCR primers for detection. (C) An example of TRAPPC12 deleted sequence. (D) Detection of TRAPPC12 protein from the indicated clones by immunoblot. Clones 8,16, 34, and 41 were confirmed to be TRAPPC12 knockouts. (E) Genomic PCR (upper panel) and immunoblots (bottom) of TRAPPC12 candidate knockout clones of HeLa cells.

**
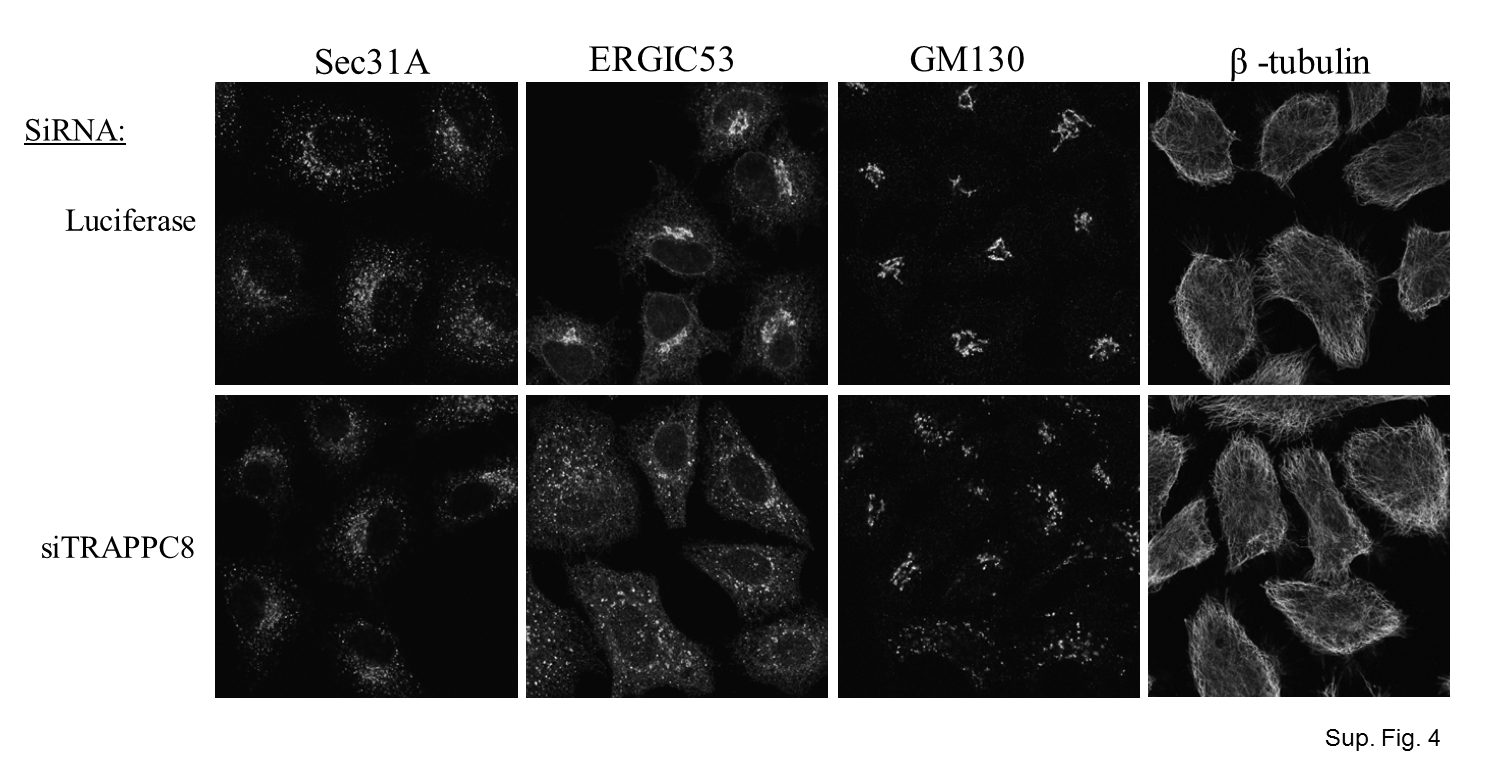
**

Supplementary Figure 4. siRNA depletion of TRAPPC8 caused dispersal of ER-Golgi Intermediate Compartments (ERGIC-53) and fragmentation of Golgi (GM130) in HEK293 cells. The integrity of the ER exit sites (Sec31A) and tubulin (β-tubulin) was not affected.


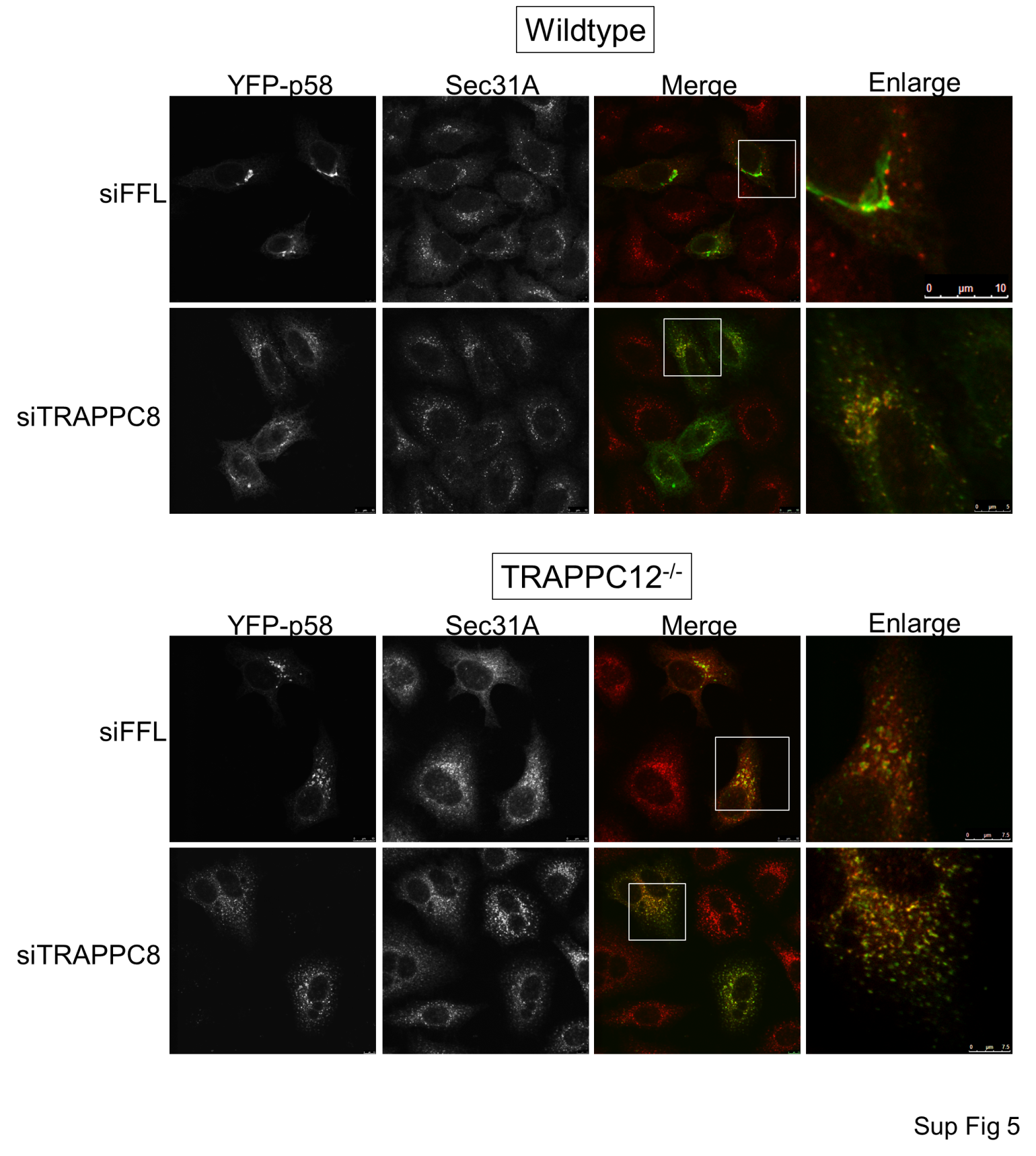


Supplementary Figure 5. ERGIC and ERES remain juxtaposed to each other in TRAPPIII defective cells. Wildtype or TRAPPC12 -/- HeLa cells were transfected with siRNA targeting TRAPPC8 (or targeting firefly luciferase, FFL, as control) for three days. Two days after siRNA depletion, the cells were further transfected with ERGIC fluorescence marker YFP-p58. Then the cells were fixed and stained with anti-Sec31A (with secondary antibody conjugated to Alexa Fluor 635) for ERES. YFP-p58 is visualized in green, and Sec31A in red in the merge images.

**
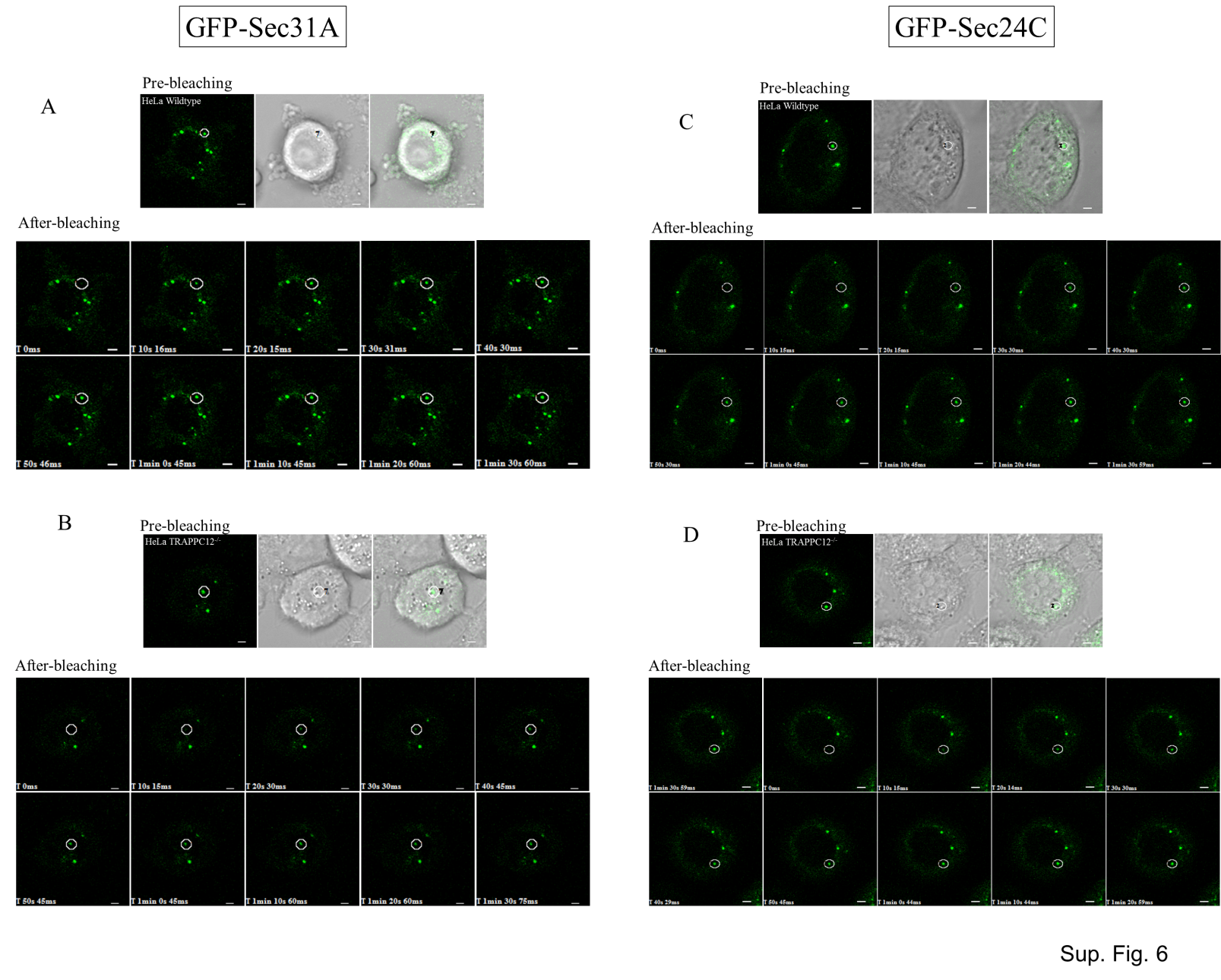
**

Supplementary Figure 6. Photobleaching of transiently transfected GFP-Sec31A or GFP-Sec24C in wild type and TRAPPC12^-/-^ HeLa cells.

A. Single plane images of a wildtype HeLa cell transiently expressed GFP-Sec31A before (upper panel) and after (lower panel) being bleached with the indicated time of fluorescence recovery. The white circle indicated the bleached region. Scale bar = 2μΜ.

B. Single plane images of a TRAPPC12^-/-^ cell transiently expressed GFP-Sec31A before (upper panel ) and after (lower panel ) being bleached.

C. Single plane images of a wildtype cell transiently expressed GFP-Sec24C before (upper panel) and after (lower panel) being bleached. The white circle indicated the bleached region. Scale bar = 2μΜ.

D. Single plane images of a TRAPPC12^-/-^ HeLa cell transiently expressed GFP-Sec24C before (upper panel ) and after (lower panel ) being bleached.
